# Promoting and sustaining a historical and global effort to prevent sepsis: the 2018 World Health Organization *SAVE LIVES: Clean Your Hands* campaign

**DOI:** 10.1186/s13054-018-2011-3

**Published:** 2018-04-13

**Authors:** Romain Martischang, Daniela Pires, Sarah Masson-Roy, Hiroki Saito, Didier Pittet

**Affiliations:** 1 Infection Control Programme and WHO Collaborating Centre on Patient Safety, University of Geneva Hospitals and Faculty of Medicine, 4 Rue Gabrielle-Perret-Gentil, 1211 Geneva, Switzerland; 2 Department of Infectious Diseases, Centro Hospitalar Lisboa Norte and Faculdade de Medicina da Universidade de Lisboa, Lisbon, Portugal; 3 Département de Microbiologie et d’Infectiologie, Centre Hospitalier Affilié Universitaire Hôtel-Dieu de Lévis, Lévis, Québec Canada; 4 Infection Prevention and Control Global Unit, Department of Service Delivery and Safety, World Health Organization, Geneva, Switzerland

**Keywords:** Sepsis, Critical care, Hand hygiene, Infection prevention and control, Alcohol-based handrub, World Health Organization, Cross-infection, Healthcare-associated infection, Global health, Stakeholders, Mobilization

## Abstract

Sepsis is estimated to affect more than 30 million patients with potentially five million deaths every year worldwide. Prevention of sepsis, as well as early recognition, diagnosis and treatment, can’t be overlooked to mitigate this global public health threat. World Health Organization (WHO) promotes hand hygiene in health care through its annual global campaign, *SAVE LIVES: Clean Your Hands* campaign on 5 May every year. The 2018 campaign targets sepsis with the overall theme “It’s in your hands; prevent sepsis in health care”.

Sepsis: the word was first traced to Homer poems in ancient Greece to describe the “decomposition” (σηψις) of Hector’s body during the siege of Troy. This word has traveled through the ages with the Arab and later the European physicians to stand for “putrefaction” [1]. Recently, the Society of Critical Care Medicine and the European Society of Intensive Care Medicine convened a task force defining sepsis as a life-threatening organ dysfunction caused by a dysregulated host response to infection [2]. Globally, sepsis affects over 30 million patients with potentially five million deaths yearly [3].

Recognizing the importance of sepsis, diverse approaches were taken to alleviate this problem, including its prevention, early recognition, diagnosis, and treatment. Every 4 years, the Surviving Sepsis Campaign elaborates evidence-based guidelines to support best practices in the early management and resuscitation of patients with sepsis. Recently, the operational clinical criteria have been updated [2] to facilitate early recognition and diagnosis of sepsis. This focus is critical because the prompt and optimal management of sepsis improves patients’ outcomes. Indeed, the majority of sepsis cases are community-acquired and present to community practitioners or in the emergency department. However, about one-third occurs during or following exposure to healthcare [4], often times complicating healthcare-acquired associated infections (HAIs). Therefore, special attention should also be dedicated to the prevention of sepsis in healthcare by improving practices and reducing HAIs.

A European-wide survey gathering 947 acute care hospitals observed a prevalence of 6.0% (country range 2.3–10.8%) of patients with at least one HAI, mainly respiratory and urinary tract, surgical site, bloodstream, and gastro-intestinal infections [5]. Intensive care units (ICUs) were heavily impacted by HAIs with an estimated prevalence of 19.7% among 11,516 ICU patients [5]. The reason for such a high prevalence lays behind the coexistence of multiple risk factors, including severe patient’s underlying conditions, device-associated disruption of skin barriers, and colonization or infection with multidrug-resistant bacteria selected by high antibiotic use [6]. Importantly, more than half of HAIs were observed following invasive procedures [7]. However, the most important vehicle for cross-transmission remains healthcare worker’s (HCW) hands [8]. Infection prevention and control (IPC) good practices are therefore essential to prevent HAI. For example, central line-associated bloodstream infection remains largely preventable through the implementation of multimodal strategies [9], including the promotion of best IPC practices, and in particular hand hygiene at the most appropriate times [10].

From the time of Semmelweis (1818–1865), hand hygiene is observed as an effective measure to prevent HAI. Today, hand hygiene is an essential part of IPC recommendations [11]. However, its apparent simplicity might highly underestimate the challenges of its implementation, especially in a complex environment such as intensive care. There are multiple predictors of poor adherence with hand hygiene but time constraint is the most critical, affecting compliance in all care settings, but especially in critical care. In ICUs, both the intensity of care, which could be defined as the number of opportunities for hand hygiene per hour of patient care, and the risk of cross-transmission during such opportunities are extremely high [12]. As a consequence, when assessed in ICUs, compliance with soap and water decreased on average by 4.7% for each additional ten opportunities for hand hygiene per hour of patient care [12].

The major game-changer to hand hygiene practices improvement was the introduction of alcohol-based handrub (ABHR) and its testing in large pragmatic clinical studies [13]. The systematic recourse to ABHR for hand hygiene proved to bypass the time constraint [12]. Critical care was at the origin of the proof of concept where system change—the replacement of handwashing with soap and water by ABHR—proved to drive a major shift toward better compliance with hand hygiene among HCWs. In parallel, a dynamic evidence-based model was proposed, targeting sequential steps of patient-to-patient transmission of pathogens via HCWs’ hands [10], and promoting the derived “My Five Moments for Hand Hygiene” to monitor, promote, summarize, and feedback times for action and average compliance. Such tools are part of a multimodal strategy implemented by the World Health Organization (WHO) made of five components: system change, training and education, observation and performance feedback, workplace reminders, and hospital safety climate [14]. The strategy proved highly successful in improving good practices worldwide [15]. Multiple characteristics specific to ICUs still impede hand hygiene optimal practices. Among these are high workload, understaffing, composition of the patient zone and ICU room design, and erroneous perception of risk transmission and hand hygiene compliance by HCWs [6]. Additional strategies were developed to tackle barriers and increase compliance through bundled interventions often composed of education, enablement, training, environmental restructuring, and persuasion [14]. Altogether, these measures prevent HAI; importantly, hand hygiene best practice is part of almost all interventions.

Recognizing the role of IPC to prevent HAI, the WHO 5 May 2018 *SAVE LIVES: Clean Your Hands* campaign selected prevention of sepsis in health care as its annual theme (Fig. 1): “It’s in your hands; prevent sepsis in health care”. This campaign, launched in 2009, aims to gather every possible actor among healthcare professionals, patients, hospital administrators, politicians, as well as other stakeholders to promote a global advocacy effort for the importance of clean hands in healthcare, yearly linked to a broader issue. This international effort is joined through the registration of health facilities on a dedicated website: http://www.who.int/infection-prevention/campaigns/clean-hands/register/en/. Through this campaign, you can be part of a larger community of more than 20,000 facilities in 179 countries to cover over 12 million HCWs and five million beds. In order to support these actions, advocacy toolkits are available on the WHO website: http://www.who.int/infection-prevention/tools/hand-hygiene/en/. The 5 May 2018 call to action for HCWs, IPC and health facility leaders, ministries of health, as well as patients and patient advocacy groups are indicated in the Table 1.

**Fig. 1:**
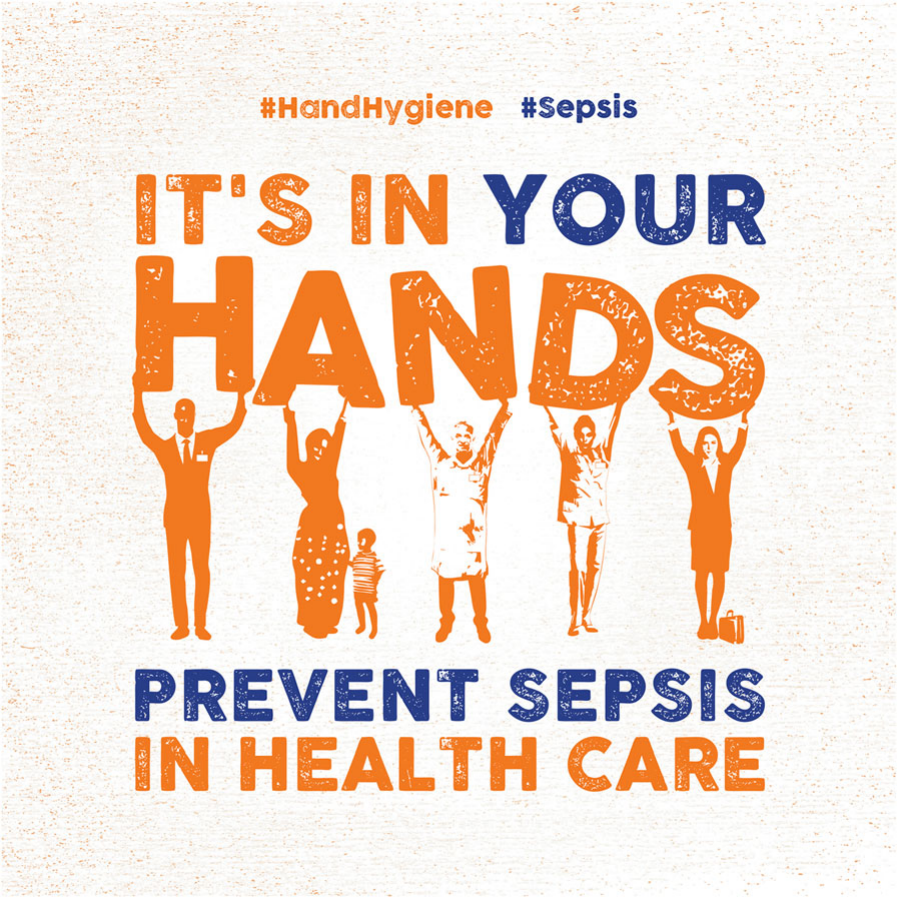
“It’s in your hands; prevent sepsis in health care”. The 5 May 2018 World Health Organization *SAVE LIVES: Clean Your Hands* campaign slogan and main promotional image (2018 hashtags: #HandHygiene #Sepsis). Campaign participants are invited to submit photos/selfies of them holding a board with the slogan and hashtags at www.CleanHandsSaveLives.org

**Table 1:** The 5 May 2018 World Health Organization *SAVE LIVES: Clean Your Hands* campaign call to action

Health workers	“Take 5 Moments* to clean your hands to prevent sepsis in health care”
IPC leaders	“Be a champion in promoting hand hygiene to prevent sepsis in health care”
Health facility leaders	“Prevent sepsis in health care, make hand hygiene a quality indicator in your hospital”
Ministries of health	“Implement the 2017 WHA sepsis resolution. Make hand hygiene a national marker of health care quality”
Patient advocacy groups	“Ask for 5 Moments* of clean hands to prevent sepsis in health care”

*Refers to the “My 5 Moments for Hand Hygiene” as published in the “WHO Guidelines on Hand Hygiene in Health Care” [10]

*IPC* Infection prevention and control, *WHA* World Health Assembly

Through the ages, the definition of sepsis has evolved, as well as its management approaches and preventive measures. Be part of this everyday promotion and advocacy to sustain what has been a historical and global effort against infections complicating healthcare. Let’s prevent sepsis resulting from patient care together.
